# Cabbage Production in West Africa and IPM with a Focus on Plant-Based Extracts and a Complementary Worldwide Vision

**DOI:** 10.3390/plants10030529

**Published:** 2021-03-11

**Authors:** Abla Déla Mondédji, Pierre Silvie, Wolali Seth Nyamador, Pierre Martin, Lakpo Koku Agboyi, Komina Amévoin, Guillaume Koffivi Ketoh, Isabelle Adolé Glitho

**Affiliations:** 1 Laboratoire d’Ecologie et d’Ecotoxicologie, Faculté des Sciences, Université de Lomé, Lomé 1 01B.P. 1515, Togo; seth.nyamador@gmail.com (W.S.N.); kamevoin@yahoo.fr (K.A.); guillaume.ketoh@gmail.com (G.K.K.); iglitho@yahoo.fr (I.A.G.); 2 CIRAD, Agroécologie et Intensification Durable Des Cultures Annuelles (AIDA), 34398 Montpellier, France; pierre.silvie@cirad.fr (P.S.); pierre.martin@cirad.fr (P.M.); 3 Institut de Recherche Pour le Développement, UMR IPME, 34AA001 Montpellier, France; 4 AIDA, CIRAD, Montpellier University, CEDEX 05, 34398 Montpellier, France; 5 CABI West Africa, PO Box CT 8630, Cantonments, Accra GA 0376800, Ghana; L.agboyi@cabi.org

**Keywords:** *Plutella xylostella*, *Hellula undalis*, *Lipaphis erysimi*, *Brevicoryne brassicae*, *Lipaphis pseudobrassicae*, pesticidal plants

## Abstract

In urban and peri-urban areas in West Africa, the cabbage *Brassica oleracea* L. (Brassicaceae) is protected using repeated high doses of synthetic insecticides. After a brief description of available IPM components, this paper presents a literature review focused on the botanical extracts that have been experimented with at the laboratory or in the field in West Africa against major cabbage pests. The literature reviewed mentions 19 plant species from 12 families used for cabbage protection in the subregion. The species most used are *Azadirachta indica*, *Capsicum frutescens*, *Ocimum gratissimum* and *Ricinus communis*. An overview of the world literature showed that a total of 13 plant species belonging to 8 families used to control cabbage pests are reported from the rest of Africa, and 140 plant species belonging to 43 families from the rest of the world. The most commonly used and tested plant species against insect pests in the three geographical areas considered is *A. indica*.

## 1. Introduction

In Africa, where vegetables are gaining ground in people’s diets, vegetable production is taking on more importance in the socioeconomic sector and sown areas are expanding. Nutritionally, vegetables improve the basic diet of populations [1], while economically and socially, their production significantly reduces unemployment [2] by giving job opportunities to a significant segment of urban dwellers [3].

Urban demand for fresh vegetables is steadily increasing [4,5]. The development of vegetable growing in urban and peri-urban areas has the advantage of bringing farmers closer to consumers while making the most efficient use of agricultural water infrastructures. In urban and peri-urban areas of Africa, vegetables are often produced on small plots.

Market gardening mostly concerns the production of vegetables. The main vegetable crops grown in the countries of the Economic Community of Western African States (ECOWAS) are lettuce (*Lactuca sativa* L.), tomato (*Solanum lycospersicum* L.), cabbages (*Brassica oleracea* L.), carrot (*Daucus carota* L.), African eggplant (*Solanum macrocarpon* L.), onion (*Allium ascalonicum* L., *Allium cepa* L.), bean (*Phaseolus vulgaris* L.), cucumber (*Cucumis sativus* L.), eggplant (*Solanum melongena* L.), beetroot (*Beta vulgaris* L.), okra (*Abelmoschus esculentus* (L.) Moench), purple amaranth (*Amaranthus blitum* L.) and jute mallow or ‘crincrin’ (*Corchorus olitorius* L.) [6,7]. Phytosanitary constraints are burdensome in the tropics, where conditions are favourable for the development and proliferation of many crop pests (insects, mites, nematodes, pathogens, etc.). In addition to the quantitative losses that could be easily assessed, there are qualitative ones related to market demand and customer perception. A damaged cabbage plant, for example, represents a loss of one kilogram of cabbage head, i.e., a mean financial loss of 300 ± 100 F CFA (0.6 ± 0.2 USD) [8]. Wholesalers, who are important middlemen in the distribution chain, usually purchase entire vegetable beds, unless elements of the bed display visible blemishes, in which case these will be excluded to avoid putting off the consumer who is focused on the appearance of vegetables [9]. Therefore, farmers rely on cheap and effective pest control methods. Chemical pest control, using synthetic pesticides, is one such method [10,11,12] and is still the most used by vegetable farmers throughout the world [13,14]. However, this crop pest management method has serious environmental drawbacks [15,16] because it results in adverse effects on useful species [17] and exposes humans and animals to serious poisoning [18,19,20,21,22]. Uncontrolled use of synthetic pesticides leads also to the development of pesticide resistance in populations of pests and pathogenic agents [23,24].

The concept of Integrated Pest Management (IPM) [25,26] nonetheless emerged more than 60 years ago and developed into a number of components such as biological pest control [27,28,29], pest-resistant cultivars [30,31,32], physical control and farming practices such as protection nets [33,34], crop diversification and companion planting [35,36], and the use of plant extracts [37,38,39,40].

The general aim of this paper is to review the situation of the protection of the round-headed cabbage *B. oleracea* L. var. capitata (Brassicacae), which is the most widely cultivated cabbage in Western Africa (ECOWAS zone) and one of the most important vegetable crops. This species is one of the main exotic leaf vegetables [41,42]. It is available all year round and commonly used in various West African recipes, in homes and restaurants alike, in particular during festivities. The substantial parasitic pressure exerted by insects and pathogens incites cabbage-farmers to make liberal use of synthetic pesticides [22,43].

This paper begins by presenting the main characteristics of the cabbage production system, showing the different elements of the context. It then lists the constraints to be dealt with, including those related to insect pests, and goes on to describe the various pest-control methods used. Brief description is given on chemical control methods using synthetic substances as well as the perception of farmers who use them and on the risks induced. Rules and regulations currently in force are summarised. The paper thereafter dwells more extensively on the diverse uses of plant extracts under trial or adopted in West Africa and presents a global review of literature on the subject.

## 2. Materials and Methods

The literature review was conducted using two main approaches. The Web of Science (WoS) database was searched with the available options (all databases between 1900 and April 2020), using as associated search words the scientific names of the main known pest species (‘*Bemisia tabaci*’, ‘*Brevicoryne brassicae*’, ’*Chrysodeixis chalcites*’, ‘*Crocidolomia binotalis*’, ‘*Helicoverpa armigera*’, ‘*Heliothis armigera*’, ‘*Hellula undalis*’, ‘*Lipaphis erysimi*’, ‘*Myzus persicae*’, ‘*Plutella xylostella*’, ‘*Trichoplusia ni*’) and the terms ‘plant extract’, ‘botanical’ and ‘cabbage’.

Since the WoS database does not include all the existing references, this first collection was completed with information found in papers, dissertations, technical information sheets and reports found via African references extracted from Google, Google Scholar, CIRAD’s Agritrop database and the Knowledge Base Knomana (KNOwledge MANAgement on pesticide plants in Africa) database currently being composed. Articles obtained from the WoS database were compiled in a Zotero library. Documents concerning the production systems, constraints faced, various control methods and existing rules and regulations were sought after in the published literature, but above all in reports, Master’s and PhD dissertations and technical datasheets, all detailed in the bibliographical references.

## 3. Results

### 3.1. Cabbage Production Systems in Urban and Peri-Urban Areas of West Africa

#### 3.1.1. Social and Demographic Characteristics of Cabbage Farmers

Social and demographic data on cabbage farmers were obtained through results of surveys on vegetable farmers in different countries of West African subregion. The ages of the farmers interviewed ranged between 15 to 80 years [44,45], with 20–50 years dominant. The proportion of women varies from sample to sample: 31.6% in Benin [46], 22.0% in Côte d’Ivoire [47], 30.9% in Ghana [46], 0% in Niger [7], 27.0% in Nigeria [45], 28.0% in Togo [44]. On average, the farmers interviewed had between 2 and 30 years of experience. Less than 50% had contact with agricultural extension services and a similar proportion had some training in vegetable production. Only about 30% took part in the activities of a vegetable farmers’ association or group. More than 50% were illiterate [7,14,45,47]. Access to information and training on the correct usage of insecticides is thus usually very limited.

#### 3.1.2. Agronomic Characteristics and Production Estimates

In West Africa, urban and peri-urban farming is usually practised close to water supply points, in swampy areas or along the littoral band, where accessing water for the crops is easier. In such areas, farmers operate within the informal economy. They cultivate plots generally less than one hectare in size and adopt practices as they see fit, without real technical recommendations or supervision. In the Sudano-Sahelian zone, in Mali (Sikasso) and Burkina Faso (Bobo Dioulasso), cabbage production is conducted on plots of 900 to 5000 m^2^ per farmer [48]. In Niger (Dosso and Zinder), cabbage is grown on areas measuring 100 to 5000 m^2^ [49,50]. Along the West African coast, access to farmland is a major constraint for the development of vegetable production, and this explains the comparatively small sizes of the planted areas. Land is often leased from private owners or belongs to state-owned domains.

Farmers usually produce cabbage alongside other vegetables, either planted in separate beds or mixed with cabbage in the same bed (intercropping, companion planting). A vegetable farmer can thus grow between three and eight species of greens simultaneously. Some produce both vegetables and ornamental plants [45]. Overall, an intensification of production is noted about three months before each country’s main festivities [42].

Quantitatively, the main vegetable crops produced in Africa in 2018 were tomatoes (20.8 million tonnes), onions (12.45 million tonnes), cabbages and other crops of the cabbage family (3.33 million tonnes), okras (3.28 million tonnes) and eggplants (2.08 million tonnes) [51]. Precise data on the statistics of cabbage production in each West African country are difficult to obtain given the poor organisation of the sector compared with industrially-produced crops for the export market such as cotton, oil palm or cocoa. It follows that for 2018 and previous years, FAOSTAT (Food and Agriculture Organization Corporate Statistical Database) data regarding ‘cabbages and other brassicas’ are available for only 5 of the 16 ECOWAS (Economic Community of West African States) countries (Table 1). 

The round-headed cabbage *B. oleracea* is not originally from the African continent. The cultivars Tropica Cross, Tropica Leader and KK-Cross, all from the same genetic pool, were genetically improved for tropical areas and, being heat-tolerant, are grown in Western and Central Africa. Cultivars such as Tropicana, Milor, Santa, Dragon, Taizé, Fabula, Tropica Cross, Tropica Leader, KK-Cross and Oxylus are all available on the market in Senegal [31]. In Togo, the cultivars most easily found on the market and planted by farmers are KK-Cross and Oxylus [38,52]. In Niger, the round-headed cabbage cultivars that can be purchased in seed shops or from travelling salesmen are Marché de Copenhague, Oxylus, Gloria, Tropica Cross, Tropica King, Vizir, KK-Cross, Leader Cross, Bandung, Gloria d’Enkhuizen, Asha, Indica, Super Comet, Fortune, King of King Cross, Nazuka and African King [53]. West African vegetable farmers do not usually produce their own seeds; most seeds are imported and produced by large international seed-farmers such as TECHNISEM.

#### 3.1.3. Production-Related Constraints

Besides the recurring problems caused by pests (insects, pathogenic nematodes, gas-tropods and rodents) and loose farm animals (goats, sheep), which feed directly on the plant, the main constraints are decreasing soil fertility, lack of workforce (for weeding, watering, transplanting), insufficient access to water, low sales or poor marketability, lack of arable land in urban areas, and land tenure and financial problems [7,42]. The impact of urbanisation on urban vegetable production is felt more or less everywhere in the West African subregion [54,55,56]. In Lomé, for example, vegetable farmers cultivate land plots wedged between dwellings and industrial infrastructures [56]. Insecure land tenure causes unstable farming conditions precluding any long-term investments (water drilling, motor pumps, etc.) that could improve crop yields. It often results in the eviction of the farmers in the face of a spreading urban development that governs how long the plots can continue being farmed. In Togo, this is a lingering issue along the coast of the costal “Maritime” region. The total surface area of land used for growing vegetables in the suburbs of Lomé thus progressively shrank from 530 ha in 2002 to 160 ha in 2014 with the urban sprawl [56]. Cabbage pests (mammals, gastropods, nematodes, insects and pathogenic fungi and viruses) are responsible for substantial crop damage. This review focuses more specifically on insect pests.

The following insect pests have been reported in cabbage production: *Plutella xylostella* L. (Lepidoptera: Plutellidae), *Hellula undalis* Fab. (Lepidoptera: Crambidae), *Spodoptera littoralis* Boisduval (Lepidoptera: Noctuidae), *Crocidolomia binotalis* Zeller (Lepidoptera: Crambidae), *Chrysodeixis chalcites* Esper (Lepidoptera: Noctuidae), *Chrysodeixis acuta* Walker (Lepidoptera: Noctuidae), *Helicoverpa* (ex *Heliothis) armigera* Hübner (Lepidoptera: Noctuidae), *Bemisia tabaci* Gennadius (Hemiptera: Aleyrodidae), *Lipaphis pseudobrassicae* Davis (Hemiptera: Aphididae), *Myzus persicae* Sulzer (Hemiptera: Aphididae), *Brevicoryne brassicae* L. (Hemiptera: Aphididae), *Lipaphis erysimi* Kaltenbach (Hemiptera: Aphididae), *Zonocerus variegatus* (L.) (Orthoptera: Pyrgomorphidae), *Phyllotreta cruciferae* Goeze (Coleoptera: Chrysomelidae) and *Jacobiasca* sp. (Hemiptera: Cicadellidae). While lepidopteran larvae gnaw the leaves, mine inside them or destroy the centre bud of young plants (such as *H. undalis*), hemipterans suck the sap of the plant. Piercing-sucking aphidid hemipterans do not create holes in leaves but suck the sap and disturb the physiology of the plant, which eventually dies.

Among the insect pests of cabbage crops in West Africa, the most feared is the diamond-back moth (*P. xylostella*). This species is present year-round, and its biological traits, high rate of reproduction, great mobility and wide range of host plants make it a formidable pest of Brassicaceae crops. It is reported from all the countries and is one of the major crucifer pests throughout the world [27,57,58,59,60]. Attacks can result in yield losses of up to 90% [61]. In Togo and Benin, losses due to ‘windowed’ and detached head leaves usually exceed 30% [62] in spite of applications of synthetic insecticide. Other insect pests such as the cabbage webworm *H. undalis*, aphids, the caterpillar of *Spodoptera littoralis*, *Chrysodeixis acuta* can significantly affect cabbage growth and head formation [11,63].

Overall, the high parasitic pressure reported by scientists and cabbage farmers hampers production stability and yield increases [64,65,66]. In Togo, damage caused by insect pests is such that some farmers along the coast have given up growing this vegetable [44,67]. Other farmers have reduced the areas planted with cabbage in order to be able to monitor infestations more closely and take better care of their plants. Since parasitic damage negatively affects the taste of the produce and the recipes’ end result, farmers feel incited to apply synthetic pesticides on their plants.

### 3.2. Use of Synthetic Pesticides in Cabbage Production and Perception of Related Risks by Farmers

Among the methods used for controlling insect pests in cabbage production, chemical control is one of the most common [14,22,46,67,68,69]. In West Africa, more than 90% of cabbage farmers use synthetic pesticides [7,44,69]; the chemical families and active ingredients used are listed in Table 2.

In West Africa, the application of active substances belonging to the major families of synthetic pesticides (synthetic pyrethroids, organophosphates, carbamates, organochlorides and avermectins) is reported [6,7,14,48,67,70,71,72,73,74,75,76,77]. Synthetic pyrethroids, organophosphates and organochlorides are the most represented. In addition to these three families of synthetic insecticides, carbamates, neonicotinoids (acetamiprid) and avermictins are also reported [7,14,67,71].

Practices regarding pesticide application are very similar in all West African countries. There are, however, some variations concerning frequency and method of application, dosage and pesticide combinations [14,22,76,78]. Pesticide use by farmers is influenced by three factors, i.e., gender, irrigation method and crop [79], but not by the pest species, as can be seen in cotton farming [80]. Cabbage is one of the vegetables most treated chemically, after tomato and onion [7]. In Burkina Faso, the mean number of phytosanitary treatments per growth cycle is 10 [81], whereas between 8 and 11 treatments are applied before harvest in Togo [43], and in Niger 16 to 18 applications (two per week) are made during the first two months of the cycle [7], with applications carried out without equipment and personal protective equipment. In Benin, in the vegetable production areas around Cotonou, cabbage farmers apply insecticides every three or four days over a period of about three months (the duration of the cycle) prior to harvest [1].

Chemicals are applied using portable pressure sprayers with backpack tanks, watering cans or various makeshift means such as whisked branches [14,22]. Most vegetable farmers of urban and peri-urban areas have access to sprayers [7], which can be purchased in nearby shops selling gardening equipment. In Bobo Dioulasso (Burkina Faso) and Sikasso (Mali), all vegetable farmers use sprayers [48].

More than 70% of farmers are unaware of the health and environmental risks involved in using synthetic pesticides [47,70]. More than 80% fail to comply with the recommended preharvest interval before harvesting vegetables, including for cabbage [7,14]. Some farmers assume that any insect observed landing in a cabbage bed is a pest. They do not know the difference between a pest insect and a beneficial insect and eliminate both. The use of synthetic pesticides at high frequencies and doses on cabbage crops destroys the environment and in particular the natural enemies of the pests, while facilitating the emergence of cases of resistance, such as seen in *P. xylostella*, to several types of insecticides [82]. It follows that cabbage farmers today find it difficult to select an insecticide because the efficacy of synthetic insecticides is currently decreasing.

Human health is another major concern for spray operators and vegetable consumers. A high proportion of farmers do not wear personal protective equipment when applying pesticides because they own none (100% in Lomé [77] and 85% in Senegal [76]).

The farming practices recorded result in an excessive use of insecticides and generate a range of related health and environmental issues. They underline the lack of perception of the hazardous nature of pesticides by the operators themselves. Pesticides not approved by the Sahelian Pesticide Committee (CSP) were easily found on farms surveyed in Niger [83]. Pesticides recorded in the field were derived from 30 active ingredients, including five (fipronil, acetochlor, dichlorvos, atrazine and paraquat dichloride) for which marketing was prohibited by CSP in the then nine member States of the Permanent Interstates Committee for Drought Control in the Sahel “Comité Inter-États de Lutte contre la Sécheresse au Sahel” (CILSS) [83,84]. Existing rules and regulations concerning the use of pesticides are general and take into account the vegetable crop sector—including cabbage production, even though it needs more scrutiny given its status as a food crop.

### 3.3. Regulations Governing Pesticide Use in West Africa

In West Africa, legislation was established at the regional level regarding management, use and control of pesticides, in compliance with WHO (World Health Organization) and FAO (Food and Agriculture Organisation) requirements and recommendations. Over the last decades, with the aim to mitigate the risks related to pesticide misuse, various protocols were signed by the leaders of regional and national communities (states) regarding the regulation of plant protection treatments in West Africa.

As regards Sahelian countries, the list of authorised pesticides is registered by the CSP of CILSS, a sub-regional organisation that comprised nine member States in 1999: Burkina Faso, Cape Verde, Chad, Gambia, Guinea-Bissau, Mali, Mauritania, Niger and Senegal [85] (now joined by Benin, Côte d’Ivoire, Guinea and Togo). Unfortunately, these regulations have not been sufficiently publicised and are poorly known by the populations of Sahelian countries, resulting in the cross-border movement of pesticides containing prohibited active substances. In addition to these regional and subregional regulations [85,86,87], there are national legislations on pest and pesticide management, such as the Decree No. 92-258 of 18 September 1992 laying down the procedure for implementing the Law No. 91-004 of 11 February 1991 regulating plant protection in the Republic of Benin [88] and the Decree No. 2018-172 of 16 May 2018 laying down the procedure for implementing the ECOWAS and “Union économique et monétaire ouest-africaine” (UEMOA) regulations concerning the registration of pesticides in the Republic of Benin [89].

Created in 1994, the CSP holds ordinary sessions twice a year in Bamako (Mali), at the Institut du Sahel, where the list of approved pesticides is published every six months. In 2012, the West African Committee for Pesticides Registration (WACPR) in charge of implementing the common regulations under the direct authority of the ECOWAS Commission was set up in Abuja (Nigeria) [87]. The mission of the WACPR is to assist the CSP in implementing the common regulation concerning the registration of pesticides within the ECOWAS region. The WACPR includes two subcommittees for greater efficiency: the Sahelian Zone subcommittee, based in Bamako and comprising seven member States (Burkina Faso, Cape Verde, Gambia, Guinea-Bissau, Mali, Niger and Senegal), and the Humid Zone sub-committee, based in Accra and comprising eight member States (Benin, Côte d’Ivoire, Ghana, Guinea, Liberia, Nigeria, Sierra Leone and Togo). Regulations are in place, but due to insufficient border controls between States, many unregistered or counterfeit pesticides with no reliable references enter the national markets. Often, information on the exact origin of dubious pesticides, with unreadable labels, are not accessible. In spite of these issues, the institutions in charge of pesticide regulation continue to pay little attention to alternative methods of crop protection, even though results obtained using agroecological approaches can be readily accessed, at least in the scientific literature. These approaches use substances that are less hazardous for human health and more respectful of the environment, but their unformulated state bars them from being submittable to CSP for official testing and registration.

### 3.4. Alternative Methods to Synthetic Chemical Insecticides

Several alternative methods exist that can reduce the use synthetic pesticides for controlling the insect pests of cabbage crops, but they are rarely used by farmers [46].

#### 3.4.1. Integrated Pest Management

Among these alternative methods, integrated pest management is a strategy that helps to reduce excessive application of synthetic insecticides [90] by integrating other practices. It was adopted in 2002 by Gambia, and supplemented with pest risk analysis and the definition of good agricultural practices [71]. This approach was identified as the best way forward at the 20th General Assembly of the Interafrican Phytosanitary Council (IAPSC) held in Côte d’Ivoire in 2002 [71]. However, very few vegetable farmers use alternative methods [46] such as the ones described below. Only 6% of them adopt alternative approaches in Benin and 3% in Ghana [46]. The different components of integrated pest control in cabbage production are detailed below.

#### 3.4.2. Selection of Improved Varieties

Glucosinolates are sulphur-containing compounds produced by cabbage plants. Fe-males of *H. undalis* are attracted to them [91] while young *P. xylostella* larvae find them highly palatable [17]. Cultivars genetically improved specifically for tropical areas (mentioned earlier) exhibit lower glucosinolate concentrations and differently-structured epicuticular wax that decrease the appeal and palatability of the leaves to adult and larval pests [92].

#### 3.4.3. Physical Pest Control and Cultural Practices

A number of farming practices can help to abate populations of cabbage pests. In the south of Benin, the use of nets as physical barriers to protect cabbage plants has shown good results against *P. xylostella* and *H. undalis* [33], better than against *S. littoralis* and the aphids *L. erysimi* and *M. persicae*. Another method is to associate cabbage plants with certain ornamentals such as «cactus queen of the right pear» or shrubs such as kola, cacao or citrus trees that act as a barrier protecting the crop. This is more easily done in places where land tenure and available space will allow it, such as in Nigeria, the States of Ogun and Oyo, in contrast to the highly urbanised environment of Lagos [45]. Regular weeding after transplanting, every two to three weeks, eliminates the weeds in which pest insects can find shelter [93]. Watering by hand (with a watering can or hose pipe) is known to alleviate *P. xylostella* infestations in cabbage [94]. Inserting some tomato or onion plants among the cabbage plants is another practice [95,96]. In such combinations (companion planting, intercropping), volatile compounds produced by other cultivated species naturally repel certain cabbage pests: garlic and onion plants, for example, emit an alliaceous compound, allyl propyl disulphide, that repels aphids [97]. In Benin, cabbage plants interspersed with the local basil, *Ocimum gratissimum* L. (Lamiaceae), were less infested with *S. littoralis*, *P. xylostella* and *H. undalis* larvae than cabbage plants in pure beds [98]. This decrease in damage permits greater yields. Crop rotation is another possible approach for farmers with at least two plots. Rotating cabbage with amaranth in order to prevent the attack of root-knot nematodes (*Meliodogyne* spp.) was reported in Benin [14]. In Togo, 21% of farmers used cultural control through crop rotations and 3% used mechanical control [67] by hand-picking and destroying lepidopteran larvae and aphids [1].

#### 3.4.4. Natural Pest Regulation and Use of Microbial Biopesticides

A number of observations concern the use of natural enemies (predatory and parasitoid insects, entomopathogenic agents, etc.) to manage the populations of cabbage pests. Many organisms found on cabbage plants parasitise or prey on insects that feed on the crop. Such organisms are called natural enemies. Most are either predators [99], parasitoids [100] or organisms pathogenic to insects [101,102,103]. Experiments have explored the potential of reinforcing these natural enemies or introducing entomopathogenic micro-organisms for biological pest control.

Predatory insects include generalist species, such as syrphids or ladybirds in the case of aphids. This particular feature makes the predation rate difficult to assess and limits the efficacy of their action over a period of time in natural conditions. Despite these difficulties, some authors have successfully identified predator species that have a controlling effect on pest populations. In Benin, the ant *Anomma nigricans* (Illiger) (Hymenoptera: Formicidae) can act as a control agent against *P. xylostella* in periurban areas [64]. The syrphid *Episyrphus balteatus* was studied in Ghana [65]. In Togo, the presence of an unidentified syrphid whose larva preys on the aphid *L. erysimi* has been reported [52].

Parasitoid insects of *P. xylostella* were closely studied in Senegal [104]. They are hymenopterans, larvae and nymphs of *Oomyzus sokolowskii* Kurdjumov (Eulophidae), larvae of *Apanteles litae* Nixon (Braconidae), *Cotesia vestalis* Haliday (Braconidae) and *Brachymeria* sp. (Chalcididae) [100]. In that country, most farmers have no knowledge of organisms active in the natural regulation of pest populations, and their agricultural practices and use of insecticides therefore stifle all chances of these beneficial insects controlling the pests [17]. The status of *Cotesia plutellae* and its response to pesticides were studied in Benin [64]. The presence of this species on cabbage production plots in a farmer field school context was also reported [52].

Also concerning *P. xylostella*, entomopathogenic fungi such as *Beauveria bassiana* and *Metarhizium anisopliae* were observed to occur naturally in Benin [103,105]. The use of *Bacillus thuringiensis* Berliner formulations such as BIOBIT™ and DIPEL™ was reported in Togo [77]. Experiments carried out on vegetable farmers’ farms in Senegal showed that organically grown cabbage protected by *B. thuringiensis* had the same yield as cabbage protected with synthetic insecticides such as dimethoate or profenofos [106]. Regarding products derived from the fermentation of the soil actinomycete bacterium *Saccharopolyspora spinosa,* a commercial formulation containing spinosyns A and D (spinosad) was tested on *P. xylostella* strains from Benin and Togo, on cabbage, in laboratory conditions [82], showing susceptibility of the pest to spinosad. However, the use by cabbage farmers of biopesticides based on micro-organisms remains low [107].

In the context of cabbage production in West Africa, where farmers face a range of constraints with few available commercial solutions for replacing—totally or in part—synthetic insecticides, plant extracts appear as a promising alternative. A bibliographical research was undertaken on this particular approach, initially centred on West Africa then broadened to the rest of the world, with a special focus on major cabbage pests.

### 3.5. Use of Botanical Extracts as an Alternative to Synthetic Pesticides in Cabbage Production

In West Africa, botanical extracts for managing the insect pests of cabbage crops in urban and peri-urban areas are prepared from local or exotic plant species or using ready-to-use formulations. In traditional methods, plant extracts are often prepared by aqueous extraction, maceration, fermentation or press-extraction, such as neem oil (*Azadirachta indica*) and castor oil (*Ricinus communis* L.). However, other solvents, such as ethanol or methanol, are also used in experiments. Essential oils can be produced by hydrodistillation. The literature was searched to gather information on the plant species and preparations employed by cabbage farmers or studied by scientists.

#### 3.5.1. Commercial Formulations

Commercial formulations of azadirachtin extracted from neem *Azadirachta indica* (Meliaceae) such as AGRONEEM^TM^, ECOZIN^TM^, AZATIN, NEEMIX^TM^, MARGOSAN O, AZATROL, NEEMBAAN, NEEMAZAL, NEEMARK, or NEEMGUARD, from eucalyptus such as BOLLCURE and others [108,109,110], are industrially manufactured in certain countries [111,112]. SUNEEM (1% EC) is employed in West Africa to control *P. xylostella* [25]. Against the aphid *L. erysimi*, neem formulations for applications in the soil are also possible, with TRINEEM for example [113]. Neem cake and *Justicia adhatoda* (= *Adhatoda vasica*) residue have been compared with mineral fertilisers [114]. Formulated extracts of plant species other than neem have also been tested, for example the BOLLCURE formulation based on *Eucalyptus* sp. leaf extracts [108] and various plant oils available on the market [115,116]. These formulations are rarely distributed in West Africa and are not sold by suppliers of synthetic pesticides in Togo for example [117]. They are not included among the plant species used or successfully tested for controlling insect pests of cabbage in West Africa.

#### 3.5.2. Plant Species Used or Successfully Tested for Controlling Insect Pests on Cabbage in West Africa

Several studies have assessed the potential of pesticidal plants for protecting cabbage in West Africa. Table 3 lists the species that have been experimentally tried and tested or used by farmers for controlling cabbage-damaging insects.

In total, 19 plant species representing 12 families have been reported: *Anacardium occidentale* L. (Anacardiaceae) [118]; three Asteraceae species (*Ageratum conyzoides* L., *Chromolaena odorata* L. and *Synedrella nodiflora* (L.) Gaertn.) [65,119,120]; one Caricaceae species (*Carica papaya* L.) [121,122,123]; two Euphorbiaceae (*Jatropha curcas* and *Ricinus communis*) [38,65,119,124]; three Lamiaceae species (*Crataeva religiosa*, *Hyptis suaveolens* and *Ocimum gratissimum*) [65,118,124,125,126,127]; one Leguminosae/Fabaceae (*Cassia sophera*) [65]; one Liliaceae (*Allium sativum*) [128,129]; one Meliaceae (*Azadirachta indica*) [25,52,62,71,93,120,121,122,123,130,131,132,133,134,135,136,137]; two Myrtaceae (*Callistemon viminalis* and *Melaleuca leucadendron*) [126]; one Poaceae (*Cymbopogon schoenanthus* (L.) Spreng.) [138]; one Rutaceae species (*Zanthoxylum xanthoxyloides*) [118] and two Solanaceae (*Capsicum frutescens* and *Nicotiana tabacum*) [65,119,128,129,139,140]. The plant parts used to prepare the different extracts are the leaves, fruits and seeds.

Neem (*A. indica*) extracts are the only ones to have been evaluated or employed against most insect pests reported for cabbage in all the countries of West Africa (Table 3), whether coastal or Sahelian, e.g., Benin [130], Côte d’Ivoire [116], Gambia [71], Ghana [93,120], Nigeria [131], Senegal [25,132] and Togo [52,62,122,123,133,134,135,136,137]. This species has been used in the form of aqueous extracts, hydroethanol extracts, methanol extracts and oils.

#### 3.5.3. Plant Species Used in the Rest of the World

In African countries outside West Africa, a total of 13 plant species from eight botanical families are mentioned in the literature surveyed (Table 4).

Comparing Table 3 and Table 4 makes it possible to identify additional botanical families, i.e., Capparaceae, Verbenaceae and Zingiberaceae [141,142,143,144,145,146,147,148,149,150,151,152], which could make interesting candidates for testing in West Africa. On the other hand, the families Anacardiaceae, Caricaceae, Euphorbiaceae, Liliaceae, Myrtaceae and Rutaceae have been tested in West Africa but not elsewhere on the continent. The species *Melia azedarach* (Meliaceae) has been successfully tested alongside neem (*A. indica*), which is also employed in West Africa.

As in West Africa, different parts of the plants are used depending on the species (leaves, stems, fruits, seeds, rhizomes). Different types of extracts have been experimented: pressed oils, essential oils (obtained by hydrodistillation), aqueous extracts, ethanol extracts, methanol extracts, petroleum ether extracts, chloroform extracts, and aqueous methanol and acetone extracts.

In West African countries, the insect cabbage pests most targeted by plant extract treatments are the lepidopterans *P. xylostella* and *H. undalis* and the aphids *L. erysimi* and *B. brassicae*. These four insects, some of which are cosmopolitan, were therefore selected to be the focus of the bibliographical research, using the references initially collected in the Zotero library. Since the aphid *L. pseudobrassicae* is regularly mentioned in research undertaken outside the African continent, it was also included in this review. With the bibliographical research method used, very few papers concerning *H. undalis* were identified from countries outside West Africa [144,153], probably because this pest is absent or less destructive in other regions or countries. Regarding aphids, a small number of references are reported concerning *L. pseudobrassicae,* in particular from Turkey [154]. Most studies found concern the aphid *L. erysimi*. The names of both species are sometimes associated [140], probably due to their morphological similarity. Most studies correspond to biological assays conducted in laboratory conditions and/or field efficacy trials of various plant extracts. A standardised method is followed, with comparisons usually made with an untreated control group, a control group treated with a synthetic insecticide, and sometimes a ‘botanical extract’ control group treated with a neem-based product.

The plant species are selected according to several criteria, which are often not detailed in the papers, e.g., use in medicine [155] or in cosmetics [156]. Most extracts are based on a single species and are aqueous, extracted at various temperatures (sometimes compared) [157,158], but some studies use combinations of two species [159,160,161]. Plant extracts are sometimes associated with an insecticidal, chemically synthetised active substance such as dimethoate [162,163], carbofuran, carbendazim or endosulfan [113]. Other experiments test extract fractions [164] or single molecules isolated from plant extracts, such as unidentified alkaloids or azadirachtin [109,155,165] or identified alkaloids such as cytisine, in this case purchased from suppliers [165]. In non-aqueous extracts, efficacy also depends on the solvent used [166,167,168].

In the rest of the world, 139 plant species representing 44 families [149,153,154,156,166,169,170,171,172,173,174,175,176,177,178,179,180,181,182,183,184,185,186,187,188,189,190,191,192,193,194,195,196,197,198,199,200,201,202,203,204,205,206,207,208,209,210,211,212,213,214,215,216,217,218,219,220,221,222,223,224] were identified (Table 5), that is, a further 31 botanical families in addition to those listed in studies from the African continent. These additional families are marked with an asterisk in Table 5. In the same botanical family, species used in Africa are mostly different from those used in the rest of the world. The only species of Caricaceae found and tested in Africa and elsewhere is *Carica papaya*, which has been studied in Pakistan to control *L. erysimi*. Among the Liliaceae, the species *Allium tuberosum* and *Veratrum nigrum* were tested in addition to *Allium sativum*, which is also employed in West Africa. With regards to Meliaceae, *Melia azedarach, Melia composita* and *Trichilia pallida* were used in experiments, in addition to *Azadirachta indica*.

The plant parts used are roughly the same as those used in West Africa, with some variations, such as stems, wood, bark, aerial parts or the whole plant. Other types of extracts are mentioned in addition to those already recorded in West Africa: cow’s milk extracts, acetone extracts, hexane extracts, petroleum ether extracts, acetic acid extracts, diethyl ether extracts and dichloromethane extracts. Most non-African studies come from Asia (Pakistan, India and China), where the aphid *L. erysimi* attacks mustard crops (*Brassica juncea*) and other *Brassica* species (*B. napus, B. parachinensis, B. rapa, B. oleracea* var. *botrytis, B. campestris* var. *tori*). 

## 4. Discussion

In the urban and peri-urban areas of Africa, cabbage is an important component of people’s diets. Cabbage heads are eaten raw or cooked. Outside leaves and those heavily perforated by insects are sometimes used to feed animals such as swine. It follows that this vegetable, when it contains traces of insecticides, can harm the health of both humans and animals [70].

Cabbage plants are highly attractive to a whole assemblage of insect pests, which mostly belong to the Lepidoptera, such as *P. xylostella, H. undalis* and *S. littoralis*, or to the group of piercing-sucking insects, such the aphids *L. erysimi* and *B. brassicae,* and the whitefly *B. tabaci*, reported as a cabbage pest by some authors in West Africa [121,139]. Of all the insect species that feed on cabbage, *P. xylostella* is the most dreaded, and moreover it attacks several species of the Brassicaceae family. This lepidopteran is therefore the object of high insecticidal control pressure. Chemical control with synthetic insecticides is the method most commonly relied on by farmers. These pesticides are often applied irrationally [225], and cabbages are sometimes harvested with no regard to the time-to-harvest waiting period. Occurrences of *P. xylostella* resistant to several families of insecticides have been documented [82,226,227,228].

Legislation on phytosanitary applications is in force in West Africa, but due to the poor control of borders and distribution channels, unauthorised pesticides inappropriate for vegetable production are found on vegetable farms.

Alternative methods to chemical control have been developed by research on pest management. One of them is physical control, using protective nets—treated with insecticides or insect repellents because *S. littoralis* can lay its eggs on untreated nets, through which the small hatchlings can later thread their way and reach the cabbage plants [229,230,231].

Augmentation biological control is another approach. It requires the rearing of large quantities of natural enemies of the targeted pest, for example parasitoid hymenopterans that parasitise *P. xylostella* [99,100]. The effectiveness of parasitoids is linked to their biological and ethological traits [232], which constitutes an additional constraint because it makes it necessary to select the best strain. Moreover, the rate of parasitism by natural enemies declines as the cabbage plants grow older, probably due to the penetration of the *P. xylostella* larvae inside the developing cabbage heads. Natural enemies are also very sensitive to repeated applications of insecticides.

All the limits mentioned for the ‘classical’ methods proposed as part of IPM plead in favour of further studies on the use of plant extracts for cabbage protection, and of the implementation of this biocontrol approach.

### 4.1. Selection of Plant Species to Be Used

Exploring the wide array of available plant species, then selecting those that appear to have the best potential for cabbage protection calls for a multi-criteria approach.

Selection criteria include some that concern plant availability, extract preparation feasibility, extract stability, extract efficacy and action mechanism. Additional criteria relate to economic aspects and environmental impacts, including unintentional impacts on non-targeted organisms such as human beings, beneficial organisms (natural enemies of the targeted pests), pollinators and earthworms, among others.

Species such as *N. tabacum* and neem *A. indica*, have long been known for their insecticidal properties against many crop pests [25,52,62,71,93,120,121,122,123,135,136,137]. They were found to dominate the census of plants used on the African continent. In Togo, like in other West African countries, extracts from the seeds or leaves of neem have proven their potency in controlling *P. xylostella*, the most destructive insect pest for cabbage crops [93,130,233]. This efficacy is also noted against other insects, such as *Zonocerus variegatus* on cabbage and *Hibiscus sabdariffa* L., in Nigeria [234] and *B. tabaci* [235]. Neem seed extracts are often combined with other pesticides, or sometimes with powdered soap, to treat vegetable crops [77]. Neem is available and even considered an invasive species [236].

Our study listed a large number of plant species, and this diversity is worth exploring. For example, *Tephrosia vogelii*, Hook leaf extract, has been tested in Zambia on larvae of both *P. xylostella* and *H. undalis*, and on the aphid *B. brassicae* [144]. In China, the efficacy of ethanol extracts of leaves of *Citrullus colocynthis* (L.), *Cannabis indica* (L.) and *Artemisia argyi* (L.) was established for controlling *B. brassicae* [194]. In Mauritius, the extracts of five plant species (*Argemone mexicana*, *Artemisia absinthium*, *Cassia occidental, Cymbopogon citratus* and *Siegesbeckia orientalis*) were shown to have an antifeedant effect on *C. binotalis* on cabbage [237]. In India, the antifeedant effect of leaf extracts of *Eucalyptus camaldulensis* and *Tylophora indica* was also proven on *H. armigera* on cabbage [238]. Among the plant species reported to be of special interest in Asia, some also occur in West Africa, often abundantly, and could be the focus of more research, in particular *Calotropis procera* (Apocynaceae) and *Lantana camara* (Verbenaceae). *Tagetes* species (Asteraceae) as well as *M. azaderach* (Meliaceae) and *T. vogelii* (Fabaceae) are other plants with an interesting potential to be tested, depending on cabbage production zones. Other unlisted extracts from plants (*Achillea millefolium* (Asteraceae), *Bidens pilosa* (Asteraceae), *Bougainvillea glabra* (Nyctaginaceae), *Chenopodium ambrosioides* (Chenopodiaceae), *Datura suaveolens* (Solanaceae), *Enterolobium contortisilliquum* (Fabaceae), *Stryphnodendron adstringens* (Fabaceae) *Mentha crispa* (Lamiaceae), *Plumbago capensis* (Plumbaginaceae), *Pothomorphe umbellate* (Piperaceae), *Sapindus saponaria* (Sapindaceae), *Solanum cernuum* (Solanaceae), *Symphytum officinale* (Boraginaceae), *Trichilia catigua* (Meliaceae) and *Ludwigia* spp (Onagraceae)) [239,240] and management tactics (microbial control, biological control, cultural control, mating disruption, insecticide rotation strategies, and plant resistance) [241] have been explored on *P. xylostella*.

The decision to experiment with certain plant species can also be based on previous knowledge regarding their chemical composition or on the known insecticidal properties of other species of the same botanical family, as in the case of *Crataeva religiosa* [125], *Cassia sophera* [65], *Callistemon viminalis* and *Melaleuca leucadendron* [126] and *Zanthoxylum xanthoxyloides* [118].

### 4.2. Factors in Favour of the Use of Botanical Extracts

Several factors appear conducive to the use of plant extracts. The plants from which extracts can be prepared on the farm are often available locally, often at no cost [242], which makes the end product cheaper than synthetic insecticides. Some of these plants are crop species, such as *A. sativum*, *Capsicum frutescens*, *C. papaya* or *O. gratissimum* [65,118,121,122,123,124,125,126,127,128,129], whose use on another food crop can therefore be considered a sensible option, guaranteeing the safety of the produce for consumers. Plant parts of other species increasingly cultivated in several African countries are easily purchased in large quantities, in particular *Anacardium occidentale* [118] and the two oil-producing Euphorbiaceae *R. communis* and *Jatropha curcas,* already reported as a promising biofuel plant [38,65,119,124]. In addition to the above, the knowledge base of the Knomana project (*Knowledge management on pesticides plants in Africa*) [243] suggests the following plant species as well: *Aloe* spp., *Capsicum annuum*, *Carica opulifolium*, *Derris elliptica*, *Eucalyptus* spp., *Lippia javanica*, *Senna siamea*, *Solanum delagoense*, *Tagetes minuta* and *T. vogelii.* Commonly-seen species sometimes even regarded as weeds also appear in our literature survey, such as *Ageratum conyzoides*, *Chromolaena odorata* and *Synedrella nodiflora* [65,119,120], *Hyptis suaveolens* [65,118,124,125,126,127], *Lantana camara* [146,147], *Ageratina adenophora* [196] or *Parthenium hysterophorus* [198].

### 4.3. Acceptability to Vegetable Farmers

As things stand, recourse to botanical extracts to manage cabbage pests varies considerably depending on the local situation. Around 40% of vegetable farmers use them in Benin [46] but only 4% in Togo [67] and Ghana [46]. Several factors can explain this. Plant extracts do not eradicate pest populations, but rather maintain their numbers below the economical injury threshold. Moreover, farmers consider that they are not practical given the time needed to prepare the extracts and the number and frequency of treatments required. High variability in subsequent yields are observed, depending on time of the year, location and sometimes on the existence of chemotypes with no impact on the targeted pests. Among many vegetable farmers, this fosters a sceptical attitude and a reluctance to adopt the practice, as has been observed in certain projects implemented in West Africa, such as the initiative ‘Potential use of plant extracts for protecting vegetable crops as an alternative to synthetic insecticides in urban and peri-urban areas’ (*Utilisation potentielle d’extraits végétaux dans la protection des cultures maraîchères comme alternative aux insecticides de synthèse en zones urbaines et périurbaines*) [244]. The lack of affordable biopesticide formulations on the market, which would compensate for the cumbersome preparation of the extracts on-farm, is still an important obstacle [46,117] that could explain the low rate of adoption of these approaches.

If affordable formulations were available, real opportunities would open up. In urban and peri-urban farms of West Africa, vegetables are often grown in fields of less than one hectare, and cabbage plots are even smaller. The small size of the cultivated plots should weigh in favour of alternative control methods.

### 4.4. Complementary Studies for the Future

More investigations are needed, in particular to facilitate the development of ready-to-use formulations, which would promote the adoption of plant extracts by vegetable farmers. Research on unintended effects must be intensified, especially regarding non-target organisms. Studies analysing the effects observed on aphids as well as on some of their natural enemies, in the laboratory or in the field, are fairly straightforward to implement. Apart from entomopathogenic fungi, not mentioned in the papers we scrutinised, the main natural enemies are predatory and parasitoid insects. The effects of the extracts were assessed by direct application on to these insects (on the imago in case of parasitoids). Regarding aphids, observations were focused on parasitoid insects such as *Diaeretiella rapae* [156,160,245] or *Aphidius gifuensis* [245], and on the predators belonging to the usual species assemblage linked to aphid colonies: chrysopids (lacewings), coccinellids (ladybirds) and syrphids (hoverflies) [109,246]. These predators are sometimes identified to species level, such as the Syrphidae *Ischiodon scutellaris* [247], the Coccinellidae *Coccinella septempunctata* [108,156], the Chrysopidae *Chrysopa carnea* [108] or the Anthocoridae bug *Orius insidiosus* [156]. Monitored pollinators are usually restricted to domestic honeybees [248,249]. Occasionally, other indicators are also monitored, such as predatory mites and springtails [113]. Different methods can be used for laboratory tests. Direct application on non-target insects is possible using Potter’s spraying tower, with aphids exposed on their host plant or without. Introducing insects at the surface of disks of leaves previously dipped in various solutions of plant extracts and air-dried constitutes another option. Indirect effects are observed when predators feed on aphids that themselves fed on plant fragments treated with plant extracts, or when parasitoid hymenopterans emerge from mummified aphids in which the parasitoid larvae were already present when the extracts were applied.

Other types of study should be undertaken both upstream (production of the plant of interest) and downstream (formulation). The stabilisation and conservation of plant extracts are thus important questions to address.

The economic viability of using botanical extracts must be comparable to that of using synthetic insecticides. Their promotion for the purpose of increasing their rate of adoption must absolutely come with specific training to improve the vegetable farmers’ current level of knowledge [46], such as organised as part of the East African initiatives ADAPPT (African Dryland Alliance for Pesticidal Plant Technologies) [250] and OPTIONs (Optimising Pesticidal Plants: Technology Innovation, Outreach and Networks) [251].

## 5. Conclusions

Vegetable farming is on the uptrend worldwide, in particular in cities. The reasons for this urbanisation are linked to the ever-increasing rural exodus. To ensure the food security of the greater part of unceasingly growing urban populations, developing vegetable farming could be the beginning of a solution. The analysis of vegetable farming systems and cabbage pest management in West African urban and peri-urban areas shows that, in spite of the limited size of farmed plots, farmers systematically use chemical control methods with no protection for the spraying operator. These synthetic insecticides are mostly organophosphates or pyrethroids, sometimes organochlorides, carbamates or avermectins. They are applied to control several insect pests, the most important of which are the diamond-back moth *P. xylostella*, the cabbage webworm *H. undalis* and several aphid species. The development of novel, more ecologically benign crop protection means would open the way for alternatives to synthetic insecticides and alleviate their negative impact on the environment.

Among the considered alternatives, the use of plant extracts appears as one of the most easily implementable options. Many studies have been and are still conducted by researchers, in the laboratory, in experimental stations and in farmers’ fields, with encouraging results that prove the efficacy of plant extracts for managing insect pests. However, with the sector still unorganised in West Africa, the adoption of botanical extracts by a majority of vegetable farmers is taking time. Each agroecological zone being unique, local intervention would be the most appropriate approach. The use of synthetic pesticides is a trend that seems unlikely to reverse in the near future in vegetable production, in particular regarding cabbage, which is an important food for the local populations. However, medium- and long-term strategies should be strengthened, calling for considerable efforts in matters of research, outreach and awareness-raising in order to foster the adoption of plant extracts and organically grown vegetables in West Africa. Cabbage farmers are in need of support tools and information to develop a more agroecological cabbage production in which pests are managed with plant extracts. Scientists, technicians, extension workers, vegetable farmers, decision-makers, business-makers, and consumers must all invest some thought and find ways to make the most of the knowledge available on the use of plant extracts. Functional networking could be an option to promote for solving this problem, which concerns public health and food security.

## Figures and Tables

**Table 1:** Production of cabbages and other Brassicaceae per ECOWAS country (FAOSTAT, 2018) (consulted 06/04/2020).

Countries	Hectares Harvested	Production in Tonnes
Benin	Non available	Non available
Burkina Faso	Non available	Non available
Cape Verde	Non available	Non available
Côte d’Ivoire	Non available	Non available
Gambia	Non available	Non available
Ghana	Non available	Non available
Guinea	Non available	Non available
Guinea-Bissau	Non available	Non available
Liberia	Non available	Non available
Mali	4.332	80.183
Mauritania	12.704	346.686
Niger	12.704	346.686
Nigeria	Non available	Non available
Senegal	3.631	63.872
Sierra Leone	Non available	Non available
Togo	3.631	55.000

**Table 2:** Main active ingredients and insecticide families used by farmers growing cabbage in urban and peri-urban areas in West Africa.

Active Ingredients/Chemical Families	Countries	References
Profenofos, Lambda-cyhalothrin, Endosulfan, Cypermethrin, Cyfluthrin, Chlorpyrifos, Deltamethrin, Acetamiprid	Benin	[14]
Organophosphates, Pyrethroids, Organochlorides	Burkina Faso	[48]
Pyrethroids, Organochlorides	Côte d’Ivoire	[70]
Organophosphates, Pyrethroids, Organochlorides, Carbamates	Gambia	[71]
Cypermethrin, Chlorpyrifos, Endosulfan, Deltamethrin, Lambda-cyhalothrin, Dimethoate	Ghana	[72]
Organophosphates, Pyrethroids, Organochlorides	Mali	[48]
Organophosphates, Pyrethroids, Avermectins	Niger	[7]
Organophosphates Organochlorides	Nigeria	[73] [74]
Dicofol, chlorpyrifos, DDT, dimethoate, Lambda-cyhalothrin Pyrethroids	Senegal	[75] [76]
Organophosphates, Pyrethroids, Carbamates, Organochlorides	Togo	[6,67,77]

**Table 3:** Plant species tested or currently used for the management of the insect pests affecting cabbage in West Africa.

Botanical Family	Plant Species	Plant part	Extract Type	Targeted Pest(s)	Country	References
Amaryllidaceae	*Allium sativum* L.	Cloves	AE	*B. brassicae, P. xylostella*	Ghana	[128]
		Cloves	AE	*B. brassicae, H. undalis, P. xylostella, T. ni*	Ghana	[129]
Anacardiaceae	*Anacardium occidentale* L.	Leaves	ME	*B. brassicae*	Ghana	[118]
Asteraceae	*Ageratum conyzoides* (L.) L.	Leaves	AE	*B. brassicae, P. xylostella*	Ghana	[65,119]
	*Chromolaena odorata* (L.) R.M.King & H.Rob.	Leaves	AE	*B. brassicae, H. undalis, P xylostella*	Ghana	[65,119,120]
	*Synedrella nodiflora* (L.) Gaertn.	Leaves	AE	*B. brassicae, P. xylostella*	Ghana	[65,119]
Caricaceae	*Carica papaya* L.	Leaves	HEE	*B. brassicae, P. xylostella*	Côte d’Ivoire	[121]
		Leaves	HEE	*L. erysimi, P. xylostella*	Togo	[122,123]
Capparaceae	*Crateva religiosa* G.Forst.	Leaves	AE	*H. undalis, P. xylostella, S. littoralis*	Senegal	[125]
Euphorbiaceae	*Jatropha curcas* L.	Leaves	AE	*B. brassicae, P. xylostella*	Ghana	[65]
	*Ricinus communis* L.	Seeds	AE	*P. xylostella*	Togo	[38]
		Seeds	oil	*P. xylostella*	Togo	[38]
		Leaves	AE	*B. brassicae, P. xylostella*	Ghana	[65]
		Seeds	AE	*H. undalis*	Côte d’Ivoire	[124]
		Leaves	AE	*P. xylostella*	Ghana	[119]
Fabaceae	*Senna sophera* (L.) Roxb.	Leaves	AE	*B. brassicae, P. xylostella*	Ghana	[65]
Lamiaceae	*Hyptis suaveolens* (L.) Poit.	Leaves	AE	*H. undalis*	Côte d’Ivoire	[124]
		Leaves	EO	*C. chalcites*	Senegal	[126]
	*Ocimum gratissimum* L.	Leaves	EO	*S. littoralis*	Côte d’Ivoire	[127]
		Leaves	AE	*B. brassicae, P. xylostella*	Ghana	[65]
		Leaves	ME	*B. brassicae*	Ghana	[118]
Meliaceae	*Azadirachta indica* A.Juss.	Seeds	AE	*P. xylostella*	Benin	[130]
		Seeds	AE	*B. brassicae, P. xylostella*	Côte d’Ivoire	[121]
		Leaves	HEE	*B. brassicae, P. xylostella*	Côte d’Ivoire	[121]
		Leaves	AE	*P. xylostella*	Gambia	[71]
		Seeds	AE	*P. xylostella*	Gambia	[71]
		Seeds	AE	*H. armigera, H. undalis, Phyllotreta cruciferae, P. xylostella, Spodoptera* sp., *Z.variegatus*	Ghana	[93]
		Seeds	oil	*C. binotalis, P. xylostella*	Nigeria	[131]
		Seeds	oil	*C. chalcites*	Senegal	[132]
		Seeds	oil	*P. xylostella*	Togo	[133,134]
		Leaves, seeds	AE	*P. xylostella, H. undalis, T. ni*	Mauritania	[25]
		Seeds	AE	*B. Brassicae, H. undalis, P xylostella*	Ghana	[120]
		Leaves	AE, HEE	*H. undalis, L. erysimi, M. persicae, P. xylostella*	Togo	[52,62,122,123,135,136,137]
Myrtaceae	*Callistemon viminalis* (Sol. ex Gaertn.) G.Don	Leaves		*C. chalcites*	Senegal	[126]
	*Melaleuca leucadendra* (L.) L.	Leaves	EO	*C. chalcites*	Senegal	[126]
Poaceae	*Cymbopogon schoenanthus* (Hochst. ex A.Rich.) Maire & Weiller	Leaves	AE EO	*P. xyslostella*	Togo	[134,138] [133,134]
Rutaceae	*Zanthoxylum zanthoxyloides* (Lam.) Zepern. & Timler	Leaves	ME	*B. brassicae*	Ghana	[118]
Solanaceae	*Capsicum annuum* L.	Fruits	AE	*B. brassicae, P. xylostella*	Ghana	[65]
		Fruits	AE	*B. brassicae, P. xylostella*	Ghana	[128]
		Fruits	AE	*B. tabaci, B. brassicae, P. xylostella*	Ghana	[139]
		Fruits	AE	*B. brassicae, H. undalis, P. xylostella, T. ni*	Ghana	[129]
		Fruits	AE	*L. erysimi, M. persicae*	Ghana	[140]
	*Nicotiana tabacum* L.	Leaves	AE	*B. brassicae, P. xylostella*	Ghana	[65]
		Leaves	AE	*P. xylostella*	Ghana	[119]

**Table 4:** Plant species tested or currently used for managing *P. xylostella*, *H. undalis* and aphids (*L. erysimi* or *B. brassicae*) in Africa outside West Africa.

Botanical Family	Plant Species	Plant Part	Extract Type	Targeted Pests	Country	References
Asteraceae	*Tithonia diversifolia* (Hemsl.) A.Gray	Leaves, stems	AE	*B. brassicae, L. erysimi, P. xylostella*	Malawi, Zambia	[141]
	*Vernonia amygdalina* Delile	Leaves, stems	AE	*B. brassicae, L. erysimi, P. xylostella*	Malawi, Zambia	[141]
	*Tagetes minuta* L.	Leaves	AE, ME, AcE, (A+M+Ac)E	*B. brassicae*	South Africa	[142]
Capparaceae	*Maerua edulis* (Gilg & Gilg-Ben.) DeWolf	Fruits	AE	*B. brassicae, P. xylostella*	Zimbabwe	[143]
Fabaceae	*Bobgunnia madagascariensis* (Desv.) J.H.Kirkbr. & Wiersema	Fruits	AE	*B. brassicae, P. xylostella*	Zimbabwe	[143]
	*Tephrosia vogelii* Hook.f.	Leaves	AE	*B. brassicae, H. undalis, P. xylostella*	Zambia	[144]
		Leaves	AE	*B. brassicae, L. erysimi, P. xylostella*	Malawi, Zambia	[141]
Lamiaceae	*Mentha piperita* L.	Leaves	AE, ME	*B. brassicae*	Ethiopia	[145]
Meliaceae	*Azadirachta indica* A.Juss.	Leaves, seeds	AE	*B. brassicae, L. erysimi, P. xylostella*	Malawi, Zambia	[141]
		Seeds	Oil	*B. brassicae, H. undalis, P. xylostella*	Zambia	[144]
		Seeds	AE	*P. xylostella*	Ethiopia	[146,147]
		Seeds	Oil	*B. brassicae, P. xylostella, L. pseudobrassicae*	Cameroon	[148]
		Seeds	AE	*P. xylostella*	Ethiopia	[149]
		Leaves	AE	*P. xylostella*	South Africa	[150]
		Fruits	ME, PEE	*B. brassicae*	Egypt	[151]
	*Melia azedarach* L.	Leaves	AE	*P. xylostella*	South Africa	[150,152]
		Leaves, seeds	AE, ME	*B. brassicae*	Ethiopia	[145]
		Fruits	ME, ChlE	*B. brassicae*	Egypt	[151]
Solanaceae	*Capsicum annuum* L.	Fruits	AE	*P. xylostella*	Ethiopia	[146,147]
Verbenaceae	*Lantana camara* L.	Leaves	AE	*P. xylostella*	Ethiopia	[146,147]
Zingiberaceae	*Curcuma longa* L.	Rhizomes	AE	*P. xylostella*	Ethiopia	[146,147]

Legend: AcE: acetone extract; AE: aqueous extract; (A+M+Ac)E: aqueous methanol and acetone extract; ChlE: chloroform extract; ME: methanol extract; oil: pressed oil; PEE: petroleum ether extract.

**Table 5:** Plant species tested or currently used for managing *P. xylostella*, *H. undalis* and aphids (*L. erysimi, B. brassicae* or *L. pseudobrassicae*) outside Africa.

Botanical Family	Plant Species	Plant Part	Extract Type	Targeted Pest(s)	Country	References
Acanthaceae *	*Justicia adhatoda* L.	Leaves & flowers	AE	*L. erysimi*	Nepal	[169]
	*Andrographis paniculate* (Burm. fil.) Nees	Whole plant	ME	*P. xylostella*	Indonesia	[170]
Acoraceae *	*Acorus calamus* L.	Leaves	AE	*L. erysimi*	Nepal	[169]
Adoxaceae *	*Sambucus nigra* L.	Leaves	AE	*B. brassicae, P. xylostella*	Poland	[171,172]
Agavaceae *	*Agave americana* L.	Leaves	CME, AE, EE	*B. brassicae*	Brazil	[173]
Amaranthaceae *	*Achyranthes aspera* L.	Leaves, stems	EE	*H. undalis*	India	[153]
	*Gomphrena globosa* L.	Seeds	EE	*P. xylostella*	Indonesia	[174]
	*Bassia scoparia* (L.) A.J.Scott	Seeds	AE, AcE, EE, EtAcE, PEE	*P. xylostella*	China	[175]
	*Achyranthes bidentata* Blume	Roots	EE	*P. xylostella*	Republic of Korea	[176]
Amaryllidaceae	*Allium sativum* L.	Bulbs	AE	*L. erysimi*	Pakistan	[177]
	*Allium tuberosum* Rottler ex Spreng.	Leaves	EO	*P. xylostella*	China	[178]
	*Veratrum nigrum* L.	Roots & rhizomes	AE, AcE, EE, EtAcE, PEE	*P. xylostella*	China	[175]
Anacardiaceae	*Schinus terebinthifolia* Raddi	Leaves	AE, ME	*P. xylostella*	Brazil	[179]
Annonaceae *	*Annona coriacea* Mart.	Leaves	AE, ME	*P. xylostella*	Brazil	[179]
	*Annona muricata* L.	Seeds	HE	*P. xylostella*	Brazil	[180]
	*Annona squamosa* L.	Seeds	oil	*B. brassicae*	India	[181]
		Seeds	AE, EE	*P. xylostella*	Indonesia	[182]
	*Duguetia furfuracea* (A.St.-Hil.) Saff.	Leaves	AE, ME	*P. xylostella*	Brazil	[179]
	*Polyalthia longifolia* (Sonn.) Thwaites	Seeds	AE, ME, ChlE, PEE	*L. erysimi*	India	[166]
Apiaceae *	*Bifora radians* M.Bieb.	Aerial parts	EO	*L. pseudobrassicae*	Turkey	[154]
	*Cicuta virosa* L.	Stems, roots	ME	*B. brassicae*	China	[183]
	*Coriandrum sativum* L.	Fruits	EO	*L. pseudobrassicae*	Turkey	[154]
	*Foeniculum vulgare* Mill.	Fruits	AlE, EO	*B. brassicae*	Brazil	[184]
		Fruits	EO	*L. pseudobrassicae*	Turkey	[154]
		Whole plant	EO	*B. brassicae*	Iran	[185]
	*Pimpinella anisum* L.	Fruits	EO	*L. pseudobrassicae*	Turkey	[154]
		Fruits	AlE, EO	*B. brassicae*	Brazil	[184]
	*Pimpinella isaurica* V.A.Matthews	Aerial parts	EO	*L. pseudobrassicae*	Turkey	[154]
	*Trachyspermum ammi* (L.) Sprague	Leaves & stems	EO	*B. brassicae*	Iran	[186]
Apocynaceae *	*Aspidosperma pyrifolium* Mart.	Peel	AE	*P. xylostella*	Brazil	[187]
		Bark	EE, AE	*P. xylostella*	Brazil	[188,189]
		Fruits	EE	*P. xylostella*	Brazil	[188]
		Roots	EE	*P. xylostella*	Brazil	[188]
	*Calotropis procera* (Aiton) Dryand.	Leaves	AE	*L. erysimi*	India	[190]
		Leaves	AE	*L. erysimi*	Pakistan	[177]
	*Cascabela thevetia* (L.) Lippold	Leaves	AE	*L. erysimi*	Pakistan	[177]
	*Catharanthus roseus* (L.) G.Don	Leaves	ME, ChlE, oil	*L. erysimi*	India	[167]
	*Tylophora indica* (Burm.f.) Merr.	Bark	AE	*L. erysimi*	India	[191]
Araceae *	*Dieffenbachia costata* Klotzsch ex Schott	Leaves	AE	*B. brassicae, P. xylostella*	Ecuador	[192]
	*Xanthosoma hylaeae* Engl. & K.Krause	Leaves	AE	*B. brassicae, P. xylostella*	Ecuador	[192]
Araliaceae *	*Panax ginseng* C.A.Mey.	Leaves & stems	ME	*P. xylostella*	China	[193]
Asphodelaceae *	*Aloe vera* (L.) Burm.f.	Leaves	EA, AcE, AcAcE, AlE	*L. erysimi*	Pakistan	[194]
Asteraceae	*Acmella oleracea* (L.) R. K. Jansen	Leaves, stems	AE, EE	*L. erysimi*	Brazil	[156]
	*Ageratina adenophora* (Spreng.) R.M.King & H.Rob.	Leaves, stems	AcE	*B. brassicae*	China	[195]
	*Artemisia argyi* H. Lév. & Vaniot	Leaves	EE	*B. brassicae*	China	[196]
	*Artemisia sieberi* Besser	Leaves & stem	HE	*B. brassicae*	Iran	[186]
	*Artemesia vulgaris* L.	Leaves	AE	*L. erysimi*	Nepal	[169]
	*Calendula officinalis* L.	Whole plant	AE	*B. brassicae, P. xylostella*	Poland	[197]
	*Chrysanthemum indicum* L.	Leaves	AE	*L. erysimi*	Pakistan	[177]
	*Clibadium* sp.	Leaves	AE	*B. brassicae, P. xylostella*	Ecuador	[192]
	*Ageratina adenophora* (Spreng.) R.M.King & H.Rob.	Leaves	AE	*L. erysimi*	India	[198]
	*Inula salsoloides* Ostenf.	Whole plant	EE	*B. brassicae, P. xylostella*	China	[199]
	*Parthenium hysterophorus* L.	Leaves, stems, flowers	PEE	*L. erysimi*	India	[200]
	*Parthenium hysterophorus* L.	Seeds	AE	*P. xylostella*	Pakistan	[201]
		Leaves	AcE, EE	*L. erysimi*	India	[168]
	*Tagetes erecta* L.	Leaves & flowers	AE	*L. erysimi*	Nepal	[169]
	*Tagetes minuta* L.	Leaves	AE	*L. erysimi*	India	[190]
		Leaves, flowers	AE	*B. brassicae*	Brazil	[202]
		Leaves & stems	EO	*B. brassicae*	Iran	[186]
	*Tanacetum parthenium* (L.) Sch.Bip.	Plant	AE	*P. xylostella*	Pakistan	[201]
Betulaceae *	*Alnus glutinosa* (L.) Gaertn.	Leaves	AE	*B. brassicae, P. xylostella*	Poland	[171,172]
Cannabaceae *	*Cannabis sativa* L.	Leaves	EE	*B. brassicae*	China	[196]
Caricaceae	*Carica papaya* L.	Leaves	AE, AcE, AcAcE, AlE	*L. erysimi*	Pakistan	[194]
Combretaceae *	*Terminalia arjuna* (Roxb. ex DC.) Wight & Arn.	Bark	AE	*L. erysimi*	India	[191]
Convolvulaceae *	*Ipomoea asarifolia* (Desr.) Roem. & Schult.	Leaves	AE	*P. xylostella*	Brazil	[189]
Cucurbitaceae *	*Citrullus colocynthis* (L.) Schrad.	Leaves	EE	*B. brassicae*	China	[196]
Cyperaceae *	*Cyperus rotundus* L.	Tubers	EE	*P. xylostella*	Indonesia	[174]
Ericaceae *	*Rhododendron molle* (Bl.) G. Don	Flowers	DCME	*P. xylostella*	China	[203]
Euphorbiaceae	*Croton jacobinensis* Baill.	Leaves, stems	EE	*P. xylostella*	Brazil	[204]
	*Croton micans* Sw.	Leaves, stems	EE	*P. xylostella*	Brazil	[204]
	*Mallotus rhamnifolius* (Willd.) Müll.Arg.	Leaves, stems	EE	*P. xylostella*	Brazil	[204]
	*Croton sellowii* Baill.	Leaves, stems	EE	*P. xylostella*	Brazil	[204]
	*Croton* sp.	Leaves	AE	*P. xylostella*	Brazil	[189]
	*Euphorbia cyparissias* L.	Whole plant	AE	*B. brassicae, P. xylostella*	Poland	[197]
	*Euphorbia tirucalli* L.	Stems	AE	*P. xylostella*	Brazil	[189]
	*Falconeria insignis* Royle	Leaves & flowers	AE	*L. erysimi*	Nepal	[169]
Fabaceae	*Pachyrhizus erosus* (L.)Urb.	Seeds	oil	*B. brassicae*	India	[181]
	*Pongamia pinnata* (L.) Pierre	Seeds	oil	*B. brassicae*	India	[181]
		Fruits	oil	*P. xylostella*	India	[205]
	*Prosopis juliflora* (Sw.) DC.	Pods	AE	*P. xylostella*	Brazil	[189]
	*Tephrosia vogelii* Hook.f.	Leaves, stems, bark, wood	AcE, ME	*P. xylostella*	China	[203]
Lamiaceae	*Ajuga nipponensis* Makino	Whole plant	AcE, ME	*P. xylostella*	China	[203]
	*Thymbra capitata* (L.) Cav.	Aerial parts	EO	*L. pseudobrassicae*	Turkey	[154]
	*Lavandula angustifolia* Mill.	Whole plant	EO	*B. brassicae*	Czech Republic	[206]
	*Mentha aquatica* L.	Leaves	EO	*L. pseudobrassicae*	Turkey	[154]
	*Mentha piperita* L.	Aerial parts	EO	*L. pseudobrassicae*	Turkey	[154]
	*Mentha pulegium* L.	Aerial parts	EO	*L. pseudobrassicae*	Turkey	[149]
	*Clinopodium serpyllifolium subsp. Fruticosum* (L.) Bräuchler	Aerial parts	EO	*L. pseudobrassicae*	Turkey	[149]
	*Nepeta cataria* L.	Leaves & stems	EO	*B. brassicae*	Iran	[186]
		Whole plant	EO	*B. brassicae*	Czech Republic	[206]
	*Origanum majorana* L.	Whole plant	EO	*B. brassicae*	Czech Republic	[206]
	*Origanum minutiflorum* O.Schwarz & P.H.Davis	Aerial parts	EO	*L. pseudobrassicae*	Turkey	[154]
	*Rosmarinus officinalis* L.	Leaves	EO	*L. pseudobrassicae*	Turkey	[154]
		Whole plant	EO	*B. brassicae*	Czech Republic	[206]
	*Salvia aramiensis* Rech.f.	Aerial parts	EO	*L. pseudobrassicae*	Turkey	[154]
	*Salvia sclarea* L.	Aerial parts	EO	*L. pseudobrassicae*	Turkey	[154]
	*Salvia tomentosa* Mill.	Aerial parts	EO	*L. pseudobrassicae*	Turkey	[154]
	*Satureja aintabensis* P.H.Davis	Aerial parts	EO	*L. pseudobrassicae*	Turkey	[154]
	*Satureja hortensis* L.	Aerial parts	EO	*L. pseudobrassicae*	Turkey	[154]
	*Satureja thymbra* L.	Aerial parts	EO	*L. pseudobrassicae*	Turkey	[154]
	*Satureja wiedemanniana* (Avé-Lall.) Velen.	Aerial parts	EO	*L. pseudobrassicae*	Turkey	[154]
	*Thymbra sintenisii* Bornm. & Azn.	Aerial parts	EO	*L. pseudobrassicae*	Turkey	[154]
	*Thymbra spicata* L.	Aerial parts	EO	*L. pseudobrassicae*	Turkey	[154]
	*Thymus carmanicus* Jalas	Whole plant	EO	*B. brassicae*	Iran	[185]
	*Zataria multiflora* Boiss.	Leaves & stems	EO	*B. brassicae*	Iran	[186]
Lauraceae *	*Cinnamomum verum* J.Presl	Whole plant	EO	*B. brassicae*	Iran	[185]
	*Laurus nobilis* L.	Whole plant	EO	*B. brassicae*	Argentina	[207]
		Leaves	EO	*L. pseudobrassicae*	Turkey	[154]
		Leaves	AE	*P. xylostella*	Brazil	[189]
Leguminosae (= Fabaceae)	*Deguelia utilis* (A.C.Sm.) A.M.G.Azevedo	Leaves	AE	*B. brassicae, P. xylostella*	Ecuador	[192]
Meliaceae	*Azadirachta indica* A.Juss.	Leaves, seeds	EE	*L. erysimi*	Bangladesh	[208]
		Leaves	AE	*L. erysimi*	Nepal	[169]
		Seeds	AE	*P. xylostella*	Brazil	[189]
		Seeds	oil	*P. xylostella*	Sri Lanka	[209]
		Seeds	AlE	*B. brassicae*	India	[210]
		Seeds	AE	*B. brassicae, P. xylostella*	Pakistan	[211]
		Leaves	AE	*L. erysimi*	India	[190]
		Seeds	AE, EE, HE	*L. erysimi*	India	[164]
		Seeds	AE	*P. xylostella*	Pakistan	[201]
		Seeds	AE	*L. erysimi*	India	[191]
		Leaves, seeds	AE, oil	*P. xylostella*	Brazil	[212]
		Seeds	ME	*P. xylostella*	China	[203]
		Seeds	AE	*P. xylostella*	Brazil	[187]
	*Melia azedarach* L.	Fruits	DEE	*P. xylostella*	Taiwan	[213]
		Seeds	ME	*P. xylostella*	India	[214]
		Fruits	AE	*P. xylostella*	Brazil	[187]
		Fruits	AE	*P. xylostella*	Brazil	[189]
	*Melia dubia* Cav.	Leaves	AE	*L. erysimi*	Nepal	[169]
	*Trichilia pallida* Sw.	Leaves, stems	AE	*P. xylostella*	Brazil	[189]
	*Trichilia silvatica* C.DC.	Leaves	AE, ME	*P. xylostella*	Brazil	[179]
Menispermaceae *	*Cissampelos glaberrima* A.St.-Hil.	Roots	AE	*P. xylostella*	Brazil	[189]
Myrtaceae	*Melaleuca citrina* (Curtis) Dum.Cours.	Leaves	ME, ChlE, oil	*L. erysimi*	India	[167]
	*Syzygium aromaticum* (L.) Merr. & L.M.Perry	Flowers	AlE, EO	*B. brassicae*	Brazil	[184]
	*Eucalyptus camaldulensis* Dehnh.	Leaves	EO	*L. pseudobrassicae*	Turkey	[154]
	*Eugenia uniflora* L.	Leaves	AE	*P. xylostella*	Brazil	[189]
	*Leptospermum petersonii* F.M.Bailey	Leaves	Oil	*P. xylostella*	Australia	[215]
	*Syzygium aromaticum* (L.) Merr. & L.M.Perry	Fruits	AE	*P. xylostella*	Brazil	[189]
		Leaves	EE	*B. brassicae*	Iran	[216]
Nitrariaceae	*Peganum harmala* L.	Seeds	EE	*P. xylostella*	Iran	[217]
Papaveraceae *	*Argemone mexicana* L.	Leaves	AE	*L. erysimi*	India	[190]
		Leaves	AcE, EE	*L. erysimi*	India	[168]
Phytolaccaceae *	*Phytolacca americana* L.	Roots	AE, AcE, EE, EtAcE PEE	*P. xylostella*	China	[175]
Pinaceae *	*Cedrus deodara* (Roxb. ex D.Don) G.Don	Wood shavings	EO	*P. xylostella*	India	[218]
	*Larix kaempferi* (Lamb.) Carrière	Root bark	AE, AcE, EE, EtAcE, PEE	*P. xylostella*	China	[175]
Piperaceae *	*Piper nigrum* L.	Fruits	ME	*P. xylostella*	Republic of Korea	[219]
Plantaginaceae *	*Digitalis purpurea* L.	Leaves	AE	*L. erysimi*	India	[191]
Poaceae *	*Cymbopogon citratus* (DC.) Stapf	Leaves	AE	*B. brassicae, P. xylostella*	Ecuador	[192]
Polygonaceae *	*Polygonum aviculare* L.	Leaves, stems	EE	*B. brassicae, P. xylostella*	China	[220]
Pontederiaceae *	*Pontederia crassipes* Mart.	Whole plant	EE	*P. xylostella*	Chicago (USA)	[221]
Rubiaceae *	*Alibertia edulis* (Rich.) A.Rich.	Leaves	AE	*P. xylostella*	Brazil	[222]
	*Alibertia intermedia*	Leaves	AE	*P. xylostella*	Brazil	[222]
	*Cordiera sessilis* (Vell.) Kuntze	Leaves	AE	*P. xylostella*	Brazil	[222]
	*Paederia foetida* L.	Leaves & stems	AE, ME, ChlE, PEE	*L. erysimi*	India	[166]
Rutaceae	*Citrus sinensis* (L.) Osbeck	Whole plant	EO	*B. brassicae*	Iran	[185]
	*Limonia acidissima* Groff	Leaves	AE, ME, ChlE, PEE	*L. erysimi*	India	[166]
Solanaceae	*Brugmansia suaveolens* (Humb. & Bonpl. ex Willd.) Sweet	Leaves, flowers, fruits	AE	*B. brassicae*	Brazil	[223]
	*Capsicum annuum* L.	Leaves, flowers, fruits	AE	*B. brassicae*	Brazil	[223]
	*Nicotiana megalosiphon* Van Heurck & Müll.Arg.	Leaves	AE	*P. xylostella*	Australia	[224]
	*Nicotiana plumbaginifolia* Viv.	Leaves	AcE, EE	*L. erysimi*	India	[168]
	*Nicotiana tabacum* L.	Leaves, flowers, fruits	AE	*B. brassicae*	Brazil	[220]
		Leaves & flowers	AE	*L. erysimi*	Nepal	[169]
	*Solanum aculeatissimum* Jacq.	Leaves, flowers, fruits	AE	*B. brassicae*	Brazil	[223]
	*Solanum pseudocapsicum* L.	Leaves, flowers, fruits	AE	*B. brassicae*	Brazil	[223]
	*Solanum bonariense* L.	Leaves, flowers, fruits	AE	*B. brassicae*	Brazil	[223]
		Leaves, flowers, fruits	AE	*B. brassicae*	Brazil	[223]
	*Solanum sisymbriifolium* Lam.	Leaves, flowers, fruits	AE	*B. brassicae*	Brazil	[223]
	*Solanum americanum* Mill.	Leaves, flowers, fruits	AE	*B. brassicae*	Brazil	[223]
	*Witheringia solanacea* L’Hér.	Leaves	AE	*B. brassicae, P. xylostella*	Ecuador	[192]
Urticaceae *	*Cecropia sp.*	Leaves	AE	*P. xylostella*	Brazil	[189]
Zingiberaceae	*Alpinia galanga* (L.) Willd.	Rhizomes	EE	*P. xylostella*	Indonesia	[174]
	*Wurfbainia compacta* (Sol. ex Maton) Skornick. & A.D.Poulsen	Fruits	EE	*P. xylostella*	Indonesia	[174]
	*Curcuma longa* L.	Rhizomes	AE, AcE, EE, EtAcE, PEE	*P. xylostella*	China	[175]
	*Elettaria cardamomum* (L.) Maton	Whole plant	EO	*B. brassicae*	Iran	[185]
Zygophyllaceae *	*Balanites aegyptiaca* (L.) Delile	Fruits	AE, ME, ChlE, PEE	*L. erysimi*	India	[166]

Legend: *: Botanical families not reportedly used in Africa (cf. Table 3 and Table 4); AcAcE: acetic acid extract; AcE: acetone extract; AE: aqueous extract; AlE: alcohol extract; ChlE: chloroform extract; CME: cow’s milk extract; DCME: dichloromethane extract; DEE: diethyl ether extract; EE: ethanol extract; EO: essential oil; EtAcE: ethyl acetate extract; HE: hexane extract; ME: methanol extract; oil: pressed oil; PEE: petroleum ether extract.

## Data Availability

Not applicable.
